# Antiflammin-2 Activates the Human Formyl-Peptide Receptor Like 1

**DOI:** 10.1100/tsw.2006.247

**Published:** 2006-10-26

**Authors:** Ahmad M. Kamal, Richard P.G. Hayhoe, Anbalakan Paramasivam, Dianne Cooper, Roderick J. Flower, Egle Solito, Mauro Perretti

**Affiliations:** ^1^ William Harvey Research Institute, Charterhouse Square, London EC1M 6BQ, UK; ^2^ Division of Neuroscience and Mental Health Imperial College London Hammersmith Hospital Campus, London W12 0NN, UK

**Keywords:** anti-inflammation, antiflammin, annexin 1, FPRL-1, formyl peptide receptors, binding, anti-inflammatory drugs, drug development, neutrophil, PMN, inflammation, lipocortin, annexin-A1, ALX, anti-inflammatory agents, peptides, FPR, signal transduction, flow chamber

## Abstract

The anti-inflammatory actions of the nonapeptide antiflammin-2, identified by homology with uteroglobin and annexin-A1 sequences, have been described in some detail, yet its mechanisms of action remain elusive. Since recent data indicate an involvement of the formyl peptide receptor (FPR)-like 1 (or FPRL-1) in the effects of annexin-A1, we have tested here the effect of antiflammin-2 with respect to this receptor family. Using HEK-293 cells expressing either human FPR and FPRL-1, and an annexin-A1 peptide as tracer ([^125^I-Tyr]-Ac2-26), we found that antiflammin-2 competed for binding only at FPRL-1, and not FPR, with an approximate EC_50_ of 1 μM. In line with data produced for the full-length protein, genuine receptor activation by antiflammin-2 was confirmed by rapid phosphorylation of extracellular-regulated kinase 1 and 2. Finally, study of the neutrophil interaction with activated endothelium under flow demonstrated an inhibitory effect of antiflammin-2, thus providing functional support to a role for the antiflammin-2/FPRL-1 anti-inflammatory axis.

### INTRODUCTION

Annexin-A1 (ANXA1), originally termed lipocortin 1, is a glucocorticoid-regulated protein with intrinsic anti-inflammatory properties. This mediator acts on several arms of the host response, however its major effects are exerted on neutrophil trafficking, activation, and life span[1,2]. It is worth noting that the antimigratory effects of ANXA1 have been reproduced by peptides derived from the N-terminal region, both *in vivo*[3,4] as well as in neutrophil/endothelium interactions in static systems *in vitro*[5,6]. In particular, a peptide termed Ac2-26 (acetyl-AMVSEFLKQAWIENEEQEYVVQTVK, Mr, 3050) was originally found to retain the antimigratory and antioedema effects of the full-length protein in several models of acute inflammation[7,8]. A new interest in the ANXA1 field has been generated by the recent observation that the protein and peptide Ac2-26 bind to a specific class of G-protein coupled 7 transmembrane receptors, the formyl-peptide receptors (FPR)[9,10].

Antiflammin-2 (AF-2) is a nonapeptide identified by sequence homology between two proteins with putative antiphospholipase $A_2$ activity, uteroglobin and ANXA1[11]. Whereas the relevance of the direct inhibition of phospholipase $A_2$ has been discussed[12], there are no doubts that AF-2, as well as its counterpart on uteroglobin termed AF-1, exerts anti-inflammatory activities when injected into recipient animals[13,14,15]. Supported by the novel notion that ANXA1 and its N-terminal–derived peptides activate members of the formyl peptide receptor (FPR) family, we questioned here whether AF-2 could also interact with these receptors. Indeed, structural analysis revealed that, in the presence of calcium ions, the AF-2 sequence would be exposed on the ANXA1 protein surface[16], therefore potentially being able to mediate or sustain protein-protein interactions. Out of three members so far described within this family, and termed FPR, FPRL-1, and FPRL-2, our analysis was restricted to the former two, since expressed by the human neutrophils[17], a cell type susceptible to AF-2 inhibitory effects[6,18].

### MATERIALS AND METHODS

#### Peptides

AF-2 (HDMNKVDK, corresponding to residues 245-254 of human ANXA1, $M_r$ 1084), the ANXA1 N-terminal peptide, Ac2-26 (AMVSEFLKQAWFIENEEQEYVQTV corresponding to residues 2-26 of human ANXA1, $M_r$ 3096), and the D-conformer of an FPRL-1-specific agonist W-peptide (WKYMVm, $M_r$ 856)[19] were prepared by the Advanced Biotechnology Centre (Imperial College School of Medicine, London) using solid-phase stepwise synthesis. In all cases, purity was more than 90% as assessed by HPLC and capillary electrophoresis (data supplied by manufacturer). The nonspecific FPR family antagonist Boc2 (N-t-butyloxycarbonyl-Phe-$_D$Leu-Phe-$_D$Leu-Phe) was obtained from ICN Pharmaceuticals (Basingstoke, Hampshire), whereas peptide WRWWWW was from Calbiochem (Darmstadt, Germany). The N-terminal–derived peptide of annexin-1, Ac2-26 was iodinated on tyrosine 21 with a specific activity of 2000 Ci/mmol ([$^{125}$I-Tyr]-Ac2-26, Amersham Biosciences, Custom Service) for subsequent binding studies.

#### Receptor Cloning

Cloning and stable expression of the human FPR and FPRL-1 receptors was performed employing standard techniques. The cDNAs encoding the open reading frame of both receptors were obtained by PCR amplification of U-937 cDNA library. PCR was performed using specific oligonucleotide primers introducing a *HindIII* restriction site immediately 5’ to the initial ATG site and a *NotI* site 3’ to the stop codon of the receptor DNA. The oligonucleotide sequences used were as follows: FPR, forward primer, 5’-GCG CAA GCT TAT GGA GAC AAA TTC CTC TCT CCC C, reverse primer, 5’-GCG CGG CCG CTC ACT TTG CCT GTA ACT CCA CCT CTG C and ALXR, forward primer, 5’-GCG CAA GCT TAT GGA AAC CAA CTT CTC CAC TCC TC, reverse primer, 5’-GCG CGG CCG CTC ACA TTG CCT GTA ACT CAG TCT CTG C). PCR products were digested, gel purified, and ligated in to pRc/CMV expression vector (Invitrogen, Paisley, Scotland) by sticky end ligation. Verification of the correct receptor construct was confirmed by sequencing. FPR and ALXR constructs were used to stably transfect HEK-293 cells using Fugene 6™ transfection reagent (Roche, Lewes, East Sussex) according to manufacturer’s instructions. Briefly, 2 μg of plasmid DNA was used to transfect HEK-293 cells ($2 \times 10^5$ cells/well in 6-well plates) cultured in supplemented Eagle’s minimum essential medium (EMEM). The medium was changed approximately 12 h later and cells left for a further 36 h prior to addition of 800 μg/ml neomycin (Invitrogen, Paisley, Scotland) to select for transfectants, which were subsequently maintained in 400 μg/ml neomycin.

### Receptor Binding Assay

Aliquots ($10^6$ cells/ml) of either FPR-HEK– or FPRL-1-HEK–transfected cells were incubated with increasing concentrations of cold Ac2-26 (0.03–50 μ*M*) and a fixed concentration (50 n*M*) of [$^{125}$I-Tyr]-Ac2-26 for 1 h in PBS supplemented with 1 m*M* $CaCl_2$ at 4°C as described[20,21]. The bound and unbound tracers were separated by filtration through Whatman GF/C glass microfibre filters using a vacuum manifold, prior to γ-counting. Identical competition experiments were performed for the other ligands, AF-2 and W-peptide.

### Assessment of ERK Activation

Activation of ERK was assessed by western blotting using specific antibodies to both phosphorylated and total ERK (Cell Signalling Technology, Hitchin, Hertfordshire). FPRL-1–transfected HEK-293 cells were seeded at 1–2 × $10^6$ cells/well in 6-well plates in supplemented EMEM for approximately 18 h. Prior to addition of agonists, the medium was changed and serum-free EMEM was added followed by addition of the relevant agonists for the reported times[21]. Cell activation was terminated by placing the cells on ice, aspirating the medium, and immediately lysing the cells in boiling lysis buffer (50 m*M* Tris-HCl pH 6.8, 1% NP-40, 2% SDS, 10% glycerol). Total protein was determined by the BCA method (Perbio Bioscience, Cramlington, Northumberland) followed by addition of 100 m*M* DTT and 0.1% bromophenol blue and heat denaturation prior to SDS-PAGE on 10% gels. Separated proteins were electrotransferred to nitrocellulose membranes (Hybond-C, Amersham Biosciences) presoaked in transfer buffer (25 m*M* Tris, 192 mM glycine, 20% methanol) at 100 mA per blot for 60 min. Membranes were blocked with 5% nonfat milk in Tris-buffered saline, pH 7.4 (50 m*M* Tris-HCl, 150 m*M* NaCl, and 0.1% Tween-20) for 60 min, followed by overnight incubation at 4°C in the relevant primary antibody (1:1000), and finally detection for 1 h with an antirabbit secondary antibody HRP-conjugate (1:2000, DakoCytomation, Ely, Cambridgeshire). Immunoreactive protein bands were detected by enhanced chemiluminescence (ECL, Amersham Biosciences). Developed Hyperfilm (Amersham Biosciences) was scanned and densitometric analysis performed with NIH Image version 1.63.

### Flow Chamber Assay

Confluent human umbilical vein endothelial cell (HUVEC; Promocell, Heidelberg, Germany) monolayers (up to passage 4) were stimulated with TNF-α (10 ng/ml; Sigma) for 4 h. Experiments with healthy volunteers were approved by the local Research Ethics Committee (P/00/029 ELCHA). Blood was collected into 3.2% sodium citrate and diluted 1:1 in RPMI 1640 (Sigma, Poole, Dorset) before separation through a double density gradient as described[22]. After neutrophil isolation and washing, contaminating erythrocytes were removed by hypotonic lysis. Neutrophils were diluted to 1 × $10^6$/ml in Dulbecco phosphate buffer saline (DPBS) supplemented with $Ca^{2+}$ and $Mg^{2+}$, and incubated with or without ANXA1 or peptide AF-2 prior to flow for 10 min at 37°C. The flow chamber was placed under a Nikon Eclipse TE3000 microscope with 100× magnification (Nikon, Inc., Melville, NY) and neutrophils (1 × $10^6$/ml) were perfused over the endothelial monolayers at a constant rate of 1 dyn/cm$^2$ using a syringe pump (Harvard Apparatus, South Natick, MA). After 8 min of perfusion, six evenly spaced fields moving in a horizontal axis were recorded for 10 sec each, using a Q-imaging Retiga EXi digital video camera (Q-imaging, Burnaby, Canada) onto a computer running Streampix capture software (Norpix Inc., Montreal, Canada). Video sequences were then loaded into ImagePro-Plus software (Media Cybernetics, Wokingham, Berkshire) for analysis. Neutrophils were manually tagged and their movements on the endothelium monitored. The total number of interacting cells was quantified, as initial cell capture, and further classified as either rolling or firmly adherent (cells that remained stationary for the 10-sec observation period) as described in the literature[23].

### Data Handling and Statistical Analysis

Experiments were performed in duplicate or triplicate and repeated at least three times. In the case of flow experiments, the six frames from each experiment were taken as a mean for comparison to repeat experiments. Within each set of experiments, where applicable, different blood donors were used for each repetition. Data are reported as mean ± SEM and statistical differences were determined by analysis of variance followed by the Dunnett's test.

## RESULTS

### Competition Binding in Transfected Cells

The ANXA1-derived nonapeptide, AF-2, and the synthetic D-conformer hexapeptide, W-peptide (WKYMVm), selectively displaced [$^{125}$-I]-Ac2-26 from FPRL-1-HEK cells (Fig. 1). Out of three distinct experiments, approximate mean $EC_{50}$ values of 1.21 μ*M* (AF-2) and 0.29 μ*M* (W-peptide), with Hill coefficients close to 1, could be calculated.

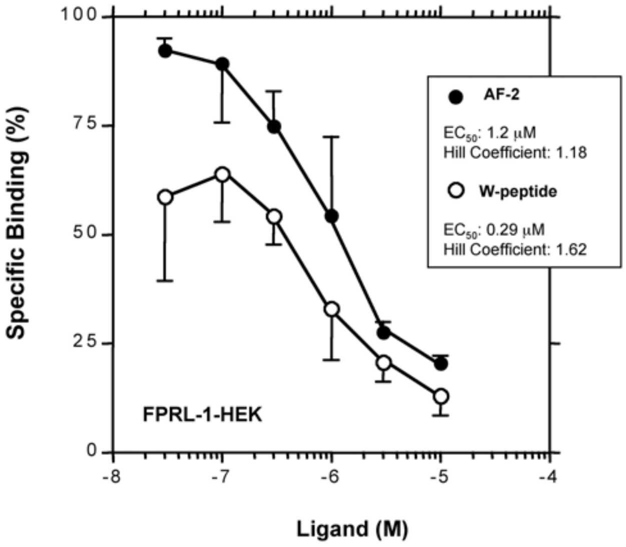

**FIGURE 1.** Competition binding with AF-2 and W-peptide ligands on FPRL-1 HEK cells**.** Competition binding curves for AF-2 (●) and W-peptide (○) in FPRL-1 HEK cells. Both peptides displaced [$^{125}$I-Tyr]-Ac2-26 tracer from binding to FPRL-1 HEK cells and approximate $EC_{50}$ and Hill constant are reported. Data presented as mean ± SEM of three independent experiments.

In contrast, neither AF-2 nor W-peptide (Figs. 2a and 2b, respectively) significantly displaced the tracer off FPR-HEK cells.

### Postreceptor Signalling

Following the demonstration of a direct physical interaction between AF-2 and W-peptide with FPRL-1, we then tested if activation of one of the major MAP kinase pathways was observed, choosing ERK kinase as this has been shown to be associated with post-FPRL-1 signalling events[21,24].

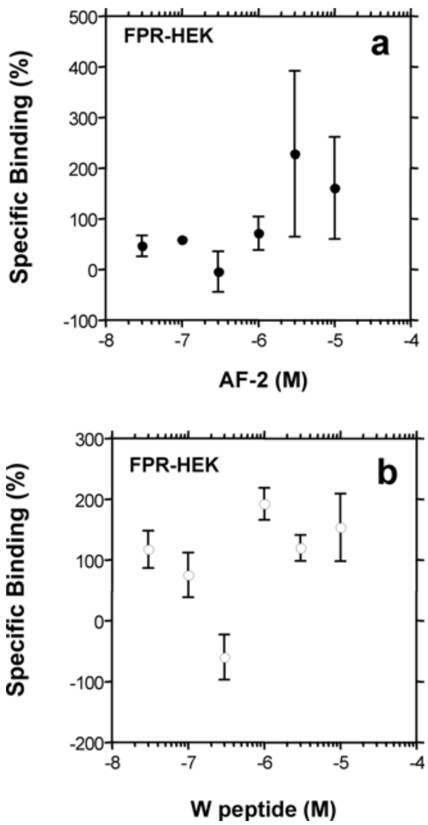

**FIGURE 2.** Lack of competition binding with AF-2 and W-peptide ligands to FPR HEK cells. Lack of displacement of the [$^{125}$I-Tyr]-Ac2-26 tracer by AF-2 (a) and W peptide (b) on FPR-HEK cells. Data presented as mean ± SEM of four independent experiments.

Incubation of AF-2 (10 μ*M*) between 2 and 30 min with FPRL-1 HEK cells resulted in a rapid time-dependent activation of ERK 1/2 (Fig. 3a). In a separate analysis, we tested all FPRL-1 agonists of the study in this assay, finding reproducible activation of this MAP kinase (Fig. 3b), by all agonists.

### Functional Studies

Finally, we tested if, by analogy to ANXA1[21], AF-2 could affect neutrophil interaction with the endothelium, a process charcaterised, and dependent on, FPRL-1 activation.

Incubation with AF-2 (10 μ*M*) produced a marked inhibition of neutrophil capture (>60%) and rolling (>80%), compared to controls, without significantly modifying the extent of neutrophil adhesion (Figs. 4a–c). The effects of AF-2 effects were concentration dependent, with a reduced degree of inhibition when lower concentrations of the anti-inflammatory peptide (1–3 μ*M*) were applied (data not shown). In addition, neutrophil incubation with the FPRL-1 selective synthetic antagonist WRWWWW (10 μ*M*) significantly prevented the effect of the maximally active concentration (10 μ*M*) of AF-2 on cell capture and rolling. This inhibitory effect was reproduced by neutrophil incubation with the pan-FPR-family receptor antagonist Boc2 (10 μ*M*).

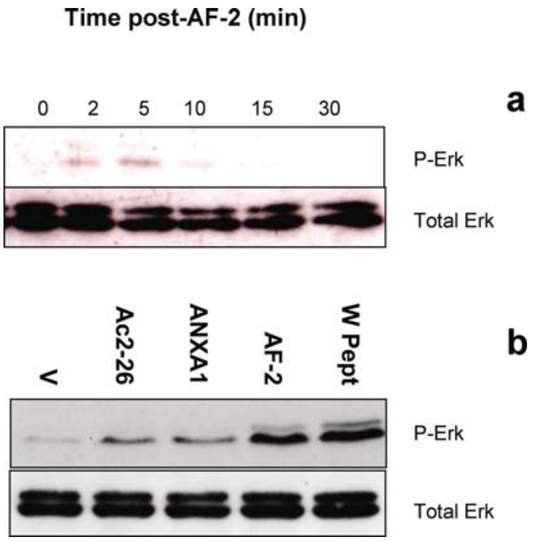

**FIGURE 3.** AF-2–induced ERK activation in FPRL-1 HEK cells**.** Panel a, time-dependent increases in ERK activation in FPRL-1-HEK cells incubated with 10 µ*M* AF-2. Panel b, ERK phosphorylation produced by FPRL-1-HEK cell incubation (10 min) with peptide Ac2-26 (10 µ*M*), ANXA1 (0.5 µ*M*), AF-2 (10 µ*M*), or W-peptide (10 µ*M*). Data are representative of two to four independent experiments.

### DISCUSSION

This study demonstrates for the first time a direct and selective interaction between the ANXA1 core-derived peptide AF-2 and the human FPRL-1 receptor. The FPRL-1-specific synthetic ligand W-peptide confirmed the selectivity of the assays employed for FPRL-1. These biochemical data are linked to functional signalling and cell-to-cell interaction analyses. We propose that this specific G-protein coupled receptor mediates at least some of the anti-inflammatory properties of AF-2.

The majority of studies investigating the role(s) of the FPR family in mediating the biological actions of ANXA1 have focussed on neutrophils[10,20,25]. However, human neutrophils are known to express both FPR and FPRL-1 receptors[17], making it difficult to dissect out the relative contribution of different receptor subtypes. A truncated N-terminal peptide of ANXA1 (residues 9-25), inhibited transendothelial neutrophil migration, calcium transients, and superoxide release in human neutrophils, effects mediated by the FPR receptor[25]. The N-terminal Ac2-26 peptide also causes calcium transients and chemotaxis of HEK-293 cells transfected with all three members of the human FPR family[10]. Our recent functional study supports this hypothesis, since in controlled *in vitro* experimental conditions, Ac2-26 bound to both FPR and FPRL-1 transfected HEK cells[21], suggesting that this molecule lacks any preassumed selectivity for a given FPR subtype.

In contrast to peptide Ac2-26, a good degree of selectivity was retained by another ANXA1-derived anti-inflammatory sequence: We showed here that AF-2 possessed selectivity for FPRL-1 in competition displacement experiments, with an $EC_{50}$ value in the low micromolar range (1.2 µ*M*). Such data reinforce the notion that the specific actions of ANXA1 might not be entirely mediated by its N-terminal region and that other sites on the molecule could be important. Interestingly, binding of AF-2 to FPRL-1 might occur with a degree of positive cooperativity as indicated by a Hill coefficient greater than 1 (1.18), which may have implications for the physicochemical and possible structure-activity relationship that this peptide may have with the receptor.

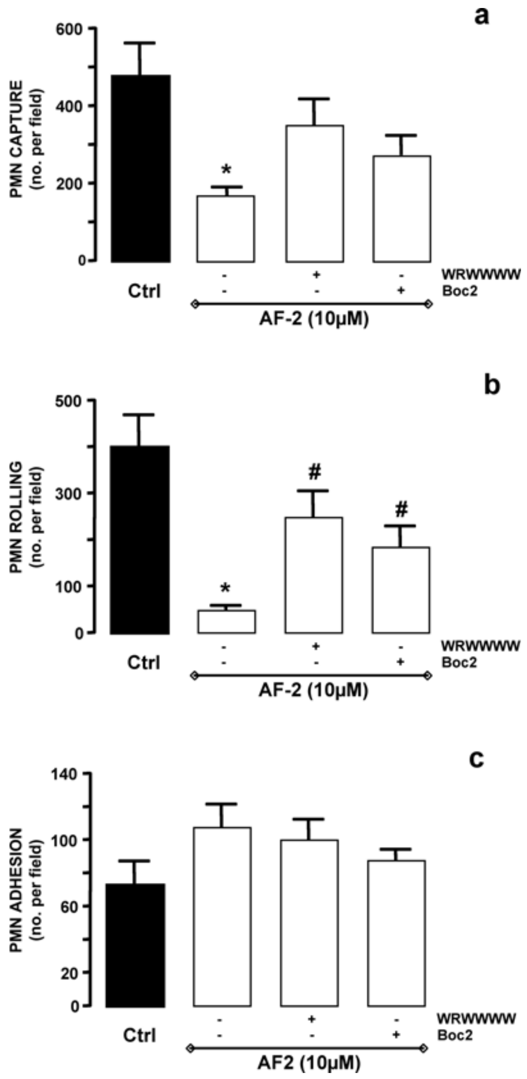

**FIGURE 4.** Effect of AF-2 on neutrophil interaction with HUVEC under flow**.** Neutrophils were incubated with 10 μ*M* AF-2 alone, with 10 μ*M* WRWWWW or with 10 μ*M* Boc2, for 10 min prior to flow over TNF-α stimulated (10 ng/ml, 4 h) HUVEC. Neutrophil-endothelium interactions were quantified off-line. (a) Number of neutrophils captured. (b) Number of rolling neutrophils. (c) Number of firmly adherent neutrophils. Data are presented as mean ± SEM of four to six independent experiments. ∗ $p < 0.05$ vs. control group; # $p < 0.05$ vs. AF-2 alone group.

In the absence of calcium, the ANXA1 N-terminal is associated with the third core domain region that is responsible for calcium binding (residues 242-257), that contains the AF-2 sequence[26]; this represents the inactive conformation of the protein[27]. However, on binding to membrane phospholipids, such as phosphatidylserine, ANXA1 affinity for calcium increases[28]. In this situation, ANXA1 changes conformation[27], possibly exposing the AF-2 sequence (third repeat; residues 247-255), which could then be potentially involved in determining biological activity or receptor binding. Interestingly, direct ANXA1 interaction with FPRL-1 occurs in the extracellular milieu, where calcium concentration is > 1 m*M*[20].

The observation that W-peptide also bound specifically to the FPRL-1 confirms a relative degree of nonspecificity for peptide ligand binding at this receptor, whose natural ligands include the antiinflammatory lipid mediator $LXA_4$[29], serum amyloid A[30], humanin[31], and ANXA1[20]. The synthetic W-peptide was employed in the present study to confirm the FPRL-1 specificity observed for AF-2[32]. W-peptide binds to both the FPRL-1 and FPRL-2 where it causes receptor phosphorylation with a calculated $K_d$ value of 0.16 μ*M*[33] within the range of what found in this study (0.29 μ*M*).

In addition to receptor binding, we show for the first time AF-2 activated FPRL-1. Similarly to what we recently reported for ANXA1[21], AF-2 addition to cells provoked transient activation of ERK-1 and ERK-2. This effect was rapid and specific since mock-transfected cells failed to demonstrate any increases in ERK activation (data not shown). Sustained ERK activation in response to constitutive overexpression of ANXA1 has been observed in RAW 264.7 macrophages and is associated with reduced cell proliferation[34,35]. Activation of this specific MAP kinase pathway has been reported for FPRL-1 and other members of this family[36,37].

The study was completed by determining the effect of AF-2 on neutrophil interaction with activated endothelial cells as monitored under flow. In line with the parent molecule[21], AF-2 inhibited this cell-to-cell response, the effect being genuinely attributable to FPRL-1, since it could be reversed by the selective antagonist WRWWWW[38]. These results are of great importance since they provide an unequivocal link between the biochemical data discussed above and the anti-inflammatory nature of this nonapeptide.

In summary, the present data illustrate for the first time selective binding of AF-2 to human FPRL-1, providing a potential mechanism of action responsible for the anti-inflammatory properties of this nonapeptide. Such data may also provide insights in to the potential active conformations of ANXA1, required to attain optimal receptor activation. The hypothesis put forward some time ago[39] in which two distinct regions of the protein (the N-terminal region and the antiflammin sequence) might be responsible for receptor binding and cooperate was reinforced by data from our functional assays.

### ACKNOWLEDGEMENTS

This work was supported by the Arthritis Research Campaign U.K. (fellowship 15755 to MP) and the Wellcome Trust (Grant 069234/Z/02). RJF is a Principal Research Fellow of the Wellcome Trust U.K.

## References

1. Perretti, M. and Flower, R.J. (2004) Annexin 1 and the biology of the neutrophil. *J. Leukoc. Biol.* **75,** 25–29.
2. Maderna, P., Yona, S., Perretti, M., and Godson, C. (2005) Modulation of phagocytosis of apoptotic neutrophils by supernatant from dexamethasone-treated macrophages and annexin-derived peptide Ac2-26. *J. Immunol.* **174,** 3727–3733.
3. Cuzzocrea, S., Tailor, A., Zingarelli, B., Salzman, A.L., Flower, R.J., Szab¢, C., and Perretti, M. (1997) Lipocortin 1 protects against splanchnic artery occlusion and reperfusion injury by affecting neutrophil migration. *J. Immunol.* **159,** 5089–5097.
4. La, M., Tailor, A., D'Amico, M., Flower, R.J., and Perretti, M. (2001) Analysis of the protection afforded by annexin 1 in ischaemia-reperfusion injury: focus on neutrophil recruitment. *Eur. J. Pharmacol.* **429,** 263–278.
5. Perretti, M., Wheller, S.K., Choudhury, Q., Croxtall, J.D., and Flower, R.J. (1995) Selective inhibition of neutrophil function by a peptide derived from lipocortin 1 N-terminus. *Biochem. Pharmacol.* **50,** 1037–1042.
6. Zouki, C., Ouellet, S., and Filep, J.G. (2000) The anti-inflammatory peptides, antiflammins, regulate the expression of adhesion molecules on human leukocytes and prevent neutrophil adhesion to endothelial cells. *FASEB J.* **14,** 572–580.
7. Cirino, G., Cicala, C., Sorrentino, L., Ciliberto, G., Arpaia, G., Perretti, M., and Flower, R.J. (1993) Anti-inflammatory actions of an N-terminal peptide from human lipocortin 1. *Br. J. Pharmacol.* **108,** 573–574.
8. Perretti, M., Ahluwalia, A., Harris, J.G., Goulding, N.J., and Flower, R.J. (1993) Lipocortin-1 fragments inhibit neutrophil accumulation and neutrophil-dependent edema in the mouse: a qualitative comparison with an anti-CD11b monoclonal antibody. *J. Immunol.* **151,** 4306–4314.
9. Perretti, M. (2003) The annexin 1 receptor(s): is the plot unravelling? *Trends Pharmacol. Sci.* **24,** 574–579.
10. Ernst, S., Lange, C., Wilbers, A., Goebeler, V., Gerke, V., and Rescher, U. (2004) An annexin 1 N-terminal peptide activates leukocytes by triggering different members of the formyl peptide receptor family. *J. Immunol.* **172,** 7669–

7676.

11. Miele, L., Cordella-Miele, E., Facchiano, A., and Mukherjee, A.B. (1988) Novel anti-inflammatory peptides from the region of highest similarity between uteroglobin and lipocortin I. *Nature* **335,** 726–730.
12. Märki, F., Pfeilschifter, J., Rink, H., and Wiesenberg, I. (1990) 'Antiflammins': two nonapeptide fragments of uteroglobin and lipocortin I have no phospholipase $A_2$-inhibitory and anti-inflammatory activity. *FEBS Lett.* **264,** 171–175.
13. Camussi, G., Tetta, C., Bussolino, F., and Baglioni, C. (1990) Antiinflammatory peptides (antiflammins) inhibit synthesis of platelet-activating factor, neutrophil aggregation and chemotaxix, and intradermal inflammatory reactions. *J. Exp. Med.* **171,** 913–927.
14. Perretti, M., Becherucci, C., Mugridge, K.G., Solito, E., Silvestri, S., and Parente, L. (1991) A novel anti-inflammatory peptide from human lipocortin 5. *Br. J. Pharmacol.* **103,** 1327–1332.
15. Moreno, J.J. (1997) Antiflammins: endogenous nonapeptide with regulatory effect on inflammation. *Gen. Pharmacol.* **28,** 23–26.
16. Weng, X., Luecke, H., Song, I.S., Kang, D.S., Kim, S.-H., and Huber, R. (1993) Crystal structure of human annexin I at 2.5å resolution. *Prot. Sci.* **2,** 448–458.
17. Le, Y., Murphy, P.M., and Wang, J.M. (2002) Formyl-peptide receptors revisited. *Trends Immunol.* **23,** 541–548.
18. Moreno, J.J. (2001) Antiflammin-2 prevents HL-60 adhesion to endothelial cells and prostanoid production induced by lipopolysaccharides. *J. Pharmacol. Exp. Ther.* **296,** 884–889.
19. Bae, Y.S., Yi, H.J., Lee, H.Y., Jo, E.J., Kim, J.I., Lee, T.G., Ye, R.D., Kwak, J.Y., and Ryu, S.H. (2003) Differential activation of formyl peptide receptor-like 1 by peptide ligands. *J. Immunol.* **171,** 6807–6813.
20. Perretti, M., Chiang, N., La, M., Fierro, I.M., Marullo, S., Getting, S.J., Solito, E., and Serhan, C.N. (2002) Endogenous lipid- and peptide-derived anti-inflammatory pathways generated with glucocorticoid and aspirin treatment activate the lipoxin $A_4$ receptor. *Nat. Med.* **8,** 1296–1302.
21. Hayhoe, R.P., Kamal, A.M., Solito, E., Flower, R.J., Cooper, D., and Perretti, M. (2006) Annexin 1 and its bioactive peptide inhibit neutrophil-endothelium interactions under flow: indication of distinct receptor involvement. *Blood* **107,** 2123–2130.
22. Perretti, M., Croxtall, J.D., Wheller, S.K., Goulding, N.J., Hannon, R., and Flower, R.J. (1996) Mobilizing lipocortin 1 in adherent human leukocytes downregulates their transmigration. *Nat. Med.* **22,** 1259–1262.
23. Patel, K.D. (1999) Mechanisms of selective leukocyte recruitment from whole blood on cytokine-activated endothelial cells under flow conditions. *J. Immunol.* **162,** 6209–6216.
24. Nanamori, M., Cheng, X., Mei, J., Sang, H., Xuan, Y., Zhou, C., Wang, M.W., and Ye, R.D. (2004) A novel nonpeptide ligand for formyl peptide receptor-like 1. *Mol. Pharmacol.* **66,** 1213–1222.
25. Walther, A., Riehemann, K., and Gerke, V. (2000) A novel ligand of the formyl peptide receptor: annexin I regulates neutrophil extravasation by interacting with the FPR. *Mol. Cell* **5,** 831–840.
26. Rosengarth, A., Rosgen, J., Hinz, H.J., and Gerke, V. (2001) Folding energetics of ligand binding proteins II. Cooperative binding of $Ca^{2+}$ to annexin I. *J. Mol. Biol.* **306,** 825–835.
27. Rosengarth, A. and Luecke, H. (2003) A calcium-driven conformational switch of the N-terminal and core domains of annexin A1. *J. Mol. Biol.* **326,** 1317–1325.
28. Schlaepfer, D.D. and Haigler, H.T. (1987) Characterization of $Ca^{2+}$-dependent phospholipid binding and phosphorylation of lipocortin I. *J. Biol. Chem.* **262,** 6931–6937.
29. Takano, T., Fiore, S., Maddox, J.F., Brady, H.R., Petasis, N.A., and Serhan, C.N. (1997) Aspirin-triggered 15-epi-lipoxin A4 (LXA4) and LXA4 stable analogues are potent inhibitors of acute inflammation: evidence for anti-inflammatory receptors. *J. Exp. Med.* **185,** 1693–1704.
30. Su, S.B., Gong, W., Gao, J.L., Shen, W., Murphy, P.M., Oppenheim, J.J., and Wang, J.M. (1999) A seven-transmembrane, G protein-coupled receptor, FPRL1, mediates the chemotactic activity of serum amyloid A for human phagocytic cells. *J. Exp. Med.* **189,** 395–402.
31. Harada, M., Habata, Y., Hosoya, M., Nishi, K., Fujii, R., Kobayashi, M., and Hinuma, S. (2004) N-Formylated humanin activates both formyl peptide receptor-like 1 and 2. *Biochem. Biophys. Res. Commun*. **324,** 255–261.
32. Dahlgren, C., Christophe, T., Boulay, F., Madianos, P.N., Rabiet, M.J., and Karlsson, A. (2000) The synthetic chemoattractant Trp-Lys-Tyr-Met-Val-DMet activates neutrophils preferentially through the lipoxin $A_4$ receptor. *Blood* **95,** 1810–1818.
33. Christophe, T., Karlsson, A., Dugave, C., Rabiet, M.J., Boulay, F., and Dahlgren, C. (2001) The synthetic peptide Trp-Lys-Tyr-Met-Val-Met-NH2 specifically activates neutrophils through FPRL1/lipoxin A4 receptors and is an agonist for the orphan monocyte-expressed chemoattractant receptor FPRL2. *J. Biol. Chem*. **276,** 21585–21593.
34. Alldridge, L.C., Harris, H.J., Plevin, R., Hannon, R., and Bryant, C.E. (1999) The annexin protein lipocortin 1 regulates the MAPK/ERK pathway. *J. Biol. Chem*. **274,** 37620–37628.
35. Alldridge, L.C. and Bryant, C.E. (2003) Annexin 1 regulates cell proliferation by disruption of cell morphology and inhibition of cyclin D1 expression through sustained activation of the ERK1/2 MAPK signal. *Exp. Cell Res.* **290,** 93–107.
36. He, R., Tan, L., Browning, D.D., Wang, J.M., and Ye, R.D. (2000) The synthetic peptide Trp-Lys-Tyr-Met-Val-D-Met is a potent chemotactic agonist for mouse formyl peptide receptor. *J. Immunol.* **165,** 4598–4605.
37. Bae, Y.S., Park, J.C., He, R., Ye, R.D., Kwak, J.Y., Suh, P.G., and Ryu, S.H. (2003) Differential signaling of formyl peptide receptor-like 1 by Trp-Lys-Tyr-Met-Val-Met-CONH2 or lipoxin A4 in human neutrophils. *Mol. Pharmacol.* **64,** 721–730.

38. Karlsson, J., Fu, H., Boulay, F., Dahlgren, C., Hellstrand, K., and Movitz, C. (2005) Neutrophil NADPH-oxidase activation by an annexin AI peptide is transduced by the formyl peptide receptor (FPR), whereas an inhibitory signal is generated independently of the FPR family receptors. *J. Leukoc. Biol.* **78,** 762–771.
39. Perretti, M. (1994) Lipocortin-derived peptides. *Biochem. Pharmacol.* **47,** 931–938.
